# Autobiographical Thinking Interferes with Episodic Memory Consolidation

**DOI:** 10.1371/journal.pone.0093915

**Published:** 2014-04-15

**Authors:** Michael Craig, Sergio Della Sala, Michaela Dewar

**Affiliations:** 1 Human Cognitive Neuroscience, Department of Psychology, University of Edinburgh, Edinburgh, United Kingdom; 2 Centre for Cognitive Ageing and Cognitive Epidemiology, University of Edinburgh, Edinburgh, United Kingdom; 3 Department of Psychology, Heriot-Watt University, Edinburgh, United Kingdom; Utrecht University, Netherlands

## Abstract

New episodic memories are retained better if learning is followed by a few minutes of wakeful rest than by the encoding of novel external information. Novel encoding is said to interfere with the consolidation of recently acquired episodic memories. Here we report four experiments in which we examined whether autobiographical thinking, i.e. an ‘internal’ memory activity, also interferes with episodic memory consolidation. Participants were presented with three wordlists consisting of common nouns; one list was followed by wakeful rest, one by novel picture encoding and one by autobiographical retrieval/future imagination, cued by concrete sounds. Both novel encoding and autobiographical retrieval/future imagination lowered wordlist retention significantly. Follow-up experiments demonstrated that the interference by our cued autobiographical retrieval/future imagination delay condition could not be accounted for by the sound cues alone or by executive retrieval processes. Moreover, our results demonstrated evidence of a temporal gradient of interference across experiments. Thus, we propose that rich autobiographical retrieval/future imagination hampers the consolidation of recently acquired episodic memories and that such interference is particularly likely in the presence of external concrete cues.

## Introduction

Quiet resting aids memory retention. People remember more newly learned episodic information if they briefly rest immediately after learning than if they attend to new external information immediately after new learning [1]–[4]. This memory benefit, which is not dependent on intentional rehearsal, is long-lasting, remaining for at least 7 days [4]. Research indicates that wakeful resting benefits episodic memory retention by enhancing memory consolidation [3]–[6]. Neuroimaging work in rodents and humans demonstrates that memory consolidation is associated with ‘offline replay’ i.e. the reactivation of recently encoded memory traces [5], [7]–[9], the magnitude of which is correlated positively with subsequent memory retention [5], [8], [9]. Importantly, offline replay occurs predominantly during periods of sleep and wakeful rest [5], [8], [9]. Sleep and wakeful rest might be especially conducive to replay because of minimal encoding of incoming external information, thereby protecting early (i.e. cellular) consolidation from encoding-related interference [4], [10].

In addition to encoding large amounts of external information, humans engage in frequent ‘internally-generated’ activities such as recalling their past and imagining their future, both of which are associated with the episodic memory system [11]–[14]. Frequently, memories of the past and imaginations about the future are triggered by external cues in our environment, especially by sensory impressions [15]. Marcel Proust [16] provides the most famous example of externally cued retrieval, describing how the taste of a madeleine cake dipped in a cup of tea led to an overwhelming deluge of memories from his childhood (p53).

Like encoding of external information, autobiographical retrieval and future imagination share some cognitive and neural networks with memory consolidation [17]–[20]. In the study reported here, we thus investigated whether these internal memory activities also interfere with the consolidation of recently acquired episodic memories.

We examined this question via four experiments in which the learning of a list of common nouns was followed by one of three 9-minute delay conditions and a subsequent surprise test of delayed word recall. In Experiments 1 and 2 the delay conditions comprised (i) wakeful resting, (ii) a picture search task (novel external encoding) and (iii) a cued retrieval/future imagination task. In Experiment 1 participants described aloud their experience in the picture search and cued retrieval/future imagination tasks. In Experiment 2 participants gave no verbal descriptions of these tasks, so as to tease apart the contribution of verbal descriptions and the tasks per se to consolidation interference. In Experiment 3 and 4 we explored the contributions of task instructions and task cues to consolidation interference.

## Experiment 1

### Method

#### Ethics statement

**Figure 1:**
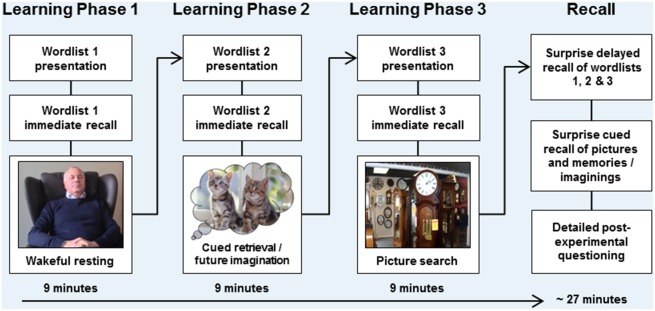
Experimental procedure. There were three wordlist learning-phases. In each learning-phase participants learned one wordlist, followed by immediate recall. Immediate recall was followed by a 9-minute delay condition, during which the critical manipulation occurred: participants either (i) rested wakefully, (ii) retrieved autobiographical memories/imagined future scenarios in response to cue sounds, or (iii) engaged in a picture search task (novel encoding). The order of the three delay conditions was counterbalanced across participants using 6 rotations (ABC, ACB, BAC, BCA, CAB, CBA), meaning that each delay condition occurred first, second and last in 12 participants each. The figure shows the example order wakeful resting → cued retrieval/future imagination → picture search. A surprise delayed-recall test for all three wordlists followed the final delay condition. In Experiment 1 participants were required to give verbal descriptions during the picture search and cued retrieval/future imagination delay conditions, whereas in Experiment 2 participants were asked to sit quietly whilst performing these delay conditions. Participants were then presented with all twenty sound cues from the picture search and cued retrieval/imagination delay conditions. For each sound they were asked to recall in as much detail as possible the associated picture, or the memory/future imagining that had been cued by each sound during the experiment. Participants also completed a structured post-experimental questionnaire that included detailed, in-depth questions of what the participants had done/thought about during each of the delay conditions, whether they had attempted to actively rehearse any material and whether they had anticipated the delayed word recall test. The person shown in this Figure has given written informed consent (as outlined in PLOS consent form) to publish his photo.

This research was approved by the University of Edinburgh’s Psychology Research Ethics Committee (Ref: 217–1112). All participants provided their informed consent in writing prior to taking part in our research. The person shown in Figure 1 has given written informed consent (as outlined in PLOS consent form) to publish his photo.

#### Participants

36 healthy young adults (11 males, 25 females; mean age = 21.6 years, *SD* = 1.40) participated in Experiment 1.

#### Procedure

We applied a within-subjects design with within-subjects factor delay condition (wakeful resting vs. picture search vs. cued retrieval/future imagination). The order of the three delay conditions was counterbalanced across participants using 6 rotations (ABC, ACB, BAC, BCA, CAB, CBA), meaning that each delay condition occurred first, second and last in 12 participants.

The to-be-retained wordlists consisted of common nouns (e.g. platform, daylight, whisky) and were matched for number of letters and syllables, frequency, familiarity, concreteness and imaginability (Kucera & Francis, 1967; British National Corpus frequency measure). Moreover, all wordlists abided to the standard phonotactic constraints of the written English language and were matched in terms of phonological neighbourhood.

As illustrated in Figure 1, the Experiment included three word-learning phases, which occurred one after the other. In each word-learning phase a recorded wordlist was presented aurally with instructions to remember as many words as possible for a subsequent immediate recall test. Immediate recall was directly followed by one of three 9-minute delay conditions, during which participants either: (i) rested wakefully, (ii) performed a picture search task or (iii) performed a cued retrieval/future imagination task.

In the 9-minute *wakeful resting* delay condition participants were asked to sit quietly in a dimly-lit testing room and relax whilst the experimenter left the room to “set up the next section of the experiment” [4].

In the 9-minute *cued retrieval/future imagination* delay condition, participants were presented with ten familiar, short (3–5 second) audible cues (e.g. a cat’s meow). The intervals between these cues varied. Participants were asked to identify each cue silently and, based on that cue, recall a past autobiographical memory *or* imagine a future autobiographical scenario. They were free to select between these, so as to replicate more closely the unrestricted autobiographical thinking in everyday life. As in previous autobiographical memory research [21]–[23] and future imagination research [24], [25], participants were requested to describe aloud their memories/future imaginations in as much vivid detail as possible while they recalled/imagined them. We used single sound cues rather than verbal sentence cues to (i) minimise verbal interference during the delay condition and (ii) make for more naturalistic memory cueing. Participants were asked to continue to describe their memories/future imaginations until interrupted by a new cue, at which point they should retrieve/imagine a new and distinctly different scenario.

In the 9-minute *picture search* delay condition participants were presented with 10 new, familiar audible cues at the same varied time intervals as in the cued retrieval/future imagination delay condition. Participants were asked to identify each cue silently. Two seconds post-cue, a detailed photo of a complex real-world scene was presented on a computer screen. Participants were required to search for items that represented or reflected the associated cue. Whilst performing this task they were asked to provide a rich verbal account of the scene, describing what they were searching for, where they were searching and any salient features that stood out to them. Each photo contained a large number of items related to the sound cue to ensure that participants continued to search until interrupted by a new cue-photo combination. We implemented audible cues and verbal descriptions in this picture search task in order to keep constant as many variables as possible across the picture search task (novel encoding) and the cued retrieval/future imagination task, the crucial difference being the internal vs. external nature of these tasks.

Upon completion of all three word-learning phases, i.e. ∼27 minutes after learning of wordlist 1, ∼18 minutes after learning of wordlist 2 and ∼9 minutes after learning wordlist 3, participants underwent a surprise delayed recall test in which they were asked to freely recall as many words as possible from all three presented wordlists (total of 45 words), in any order. We probed recall for all three wordlists together to ensure that recall would come as a surprise for all delay conditions, thus reducing the likelihood of conscious rehearsal of words during the latter delay conditions [4]. Participants were then presented with all twenty sound cues from the picture search and cued retrieval/imagination delay conditions. For each sound they were asked to recall in as much detail as possible the associated picture, or the memory/future imagining that had been cued by each sound during the experiment.

Participants also completed the Vividness of Visual Imagery Questionnaire (VVIQ) [26] and a structured post-experimental questionnaire. The latter included detailed in-depth questions of what the participants had done/thought about during each of the delay conditions, whether they had attempted to actively rehearse any material and whether they had anticipated the delayed word recall test [4].

#### Scoring

For each delay condition we computed the number of words recalled correctly at immediate recall (/15) and delayed recall (/15). In order to examine how much of the immediately recalled material was retained in each of the three delay conditions, we computed percentage retention scores for each delay condition ((delayed recall/immediate recall) ×100).

We used Levine et al.’s [23] method to score the descriptions of the past and future. Descriptions were segmented into internal (episodic) and external (semantic) details. Internal details were further categorised as ‘Event’, ‘Time’, ‘Place’, ‘Perceptual’ or ‘Thoughts/emotion’. External details were further categorised as ‘Event’, ‘Semantic’, ‘Repetition’ or ‘Other’. The number of details in each category was counted to derive a quantitative (i) internal score, (ii) external score, and (iii) total score (internal + external) of the richness of each description. We also derived a qualitative score of the richness of each description by rating each ‘internal’ category on a scale from 0–3 and adding this to the ‘episodic richness’ score (0–6), resulting in a qualitative ‘episodic re-experiencing’ score (maximum = 18) [23]. We scored the descriptions of the pictures by counting the number of key observations.

### Results

#### Immediate word recall

There was no difference between the delay conditions in immediate recall performance (*F*(2,70) = 0.054, *p* = .948, ηρ^2^ = .002), indicating that baseline memory performance was matched across the three delay conditions.

#### Wordlist retention

**Figure 2:**
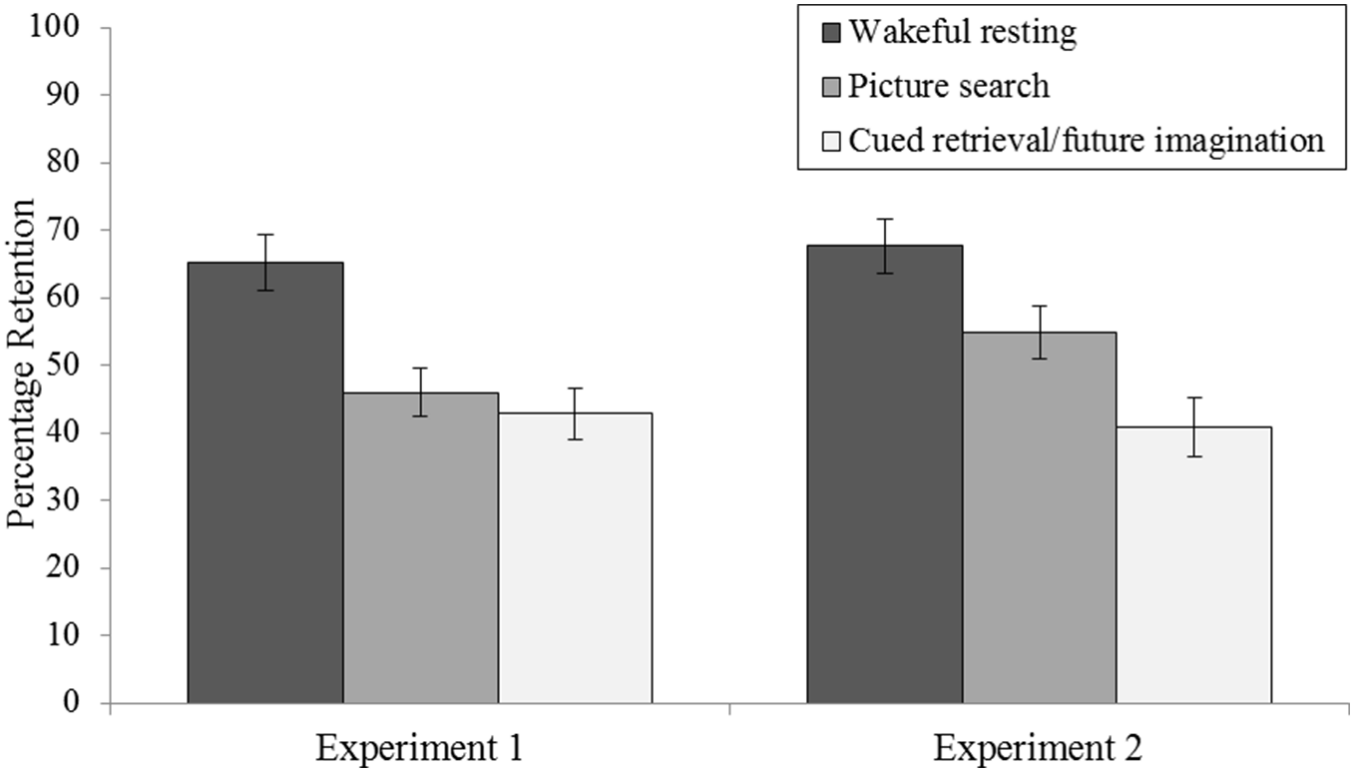
Mean percentage-retention scores as a function of delay condition (wakeful resting vs. picture search vs. cued retrieval/future imagination) in Experiment 1 (where participants provided verbal descriptions during the picture search and cued retrieval/future imagination delay conditions) and Experiment 2 (where participants sat quietly during all delay conditions). Percentage-retention scores were calculated by dividing the number of words recalled correctly in the delayed recall test by the number of words recalled correctly at immediate recall and multiplying by 100. Error bars show the standard error of the mean.

Mean percentage retention scores ((delayed recall/immediate recall) ×100) are displayed in Figure 2. A main effect of delay condition was observed (*F*(2,70) = 11.875, *p*<.001, ηρ^2^ = .253). Planned *t* tests showed that wordlist retention was significantly higher when learning was followed by wakeful rest than by the picture search (*t*(35) = 4.351, *p*<.001) or cued retrieval/future imagination (*t*(35) = 3.908, *p<*.005) delay conditions. Retention did not differ significantly between the picture search and cued retrieval/future imagination delay conditions (*t*(35) = −.653, *p* = .518).

#### Expected recall and rehearsal

Three participants expected the delayed recall and thought about wordlist material during some of the rest delay. However, none of the results changed when removing these participants from the analyses.

#### Delay condition activity and wordlist retention

The majority of participants (N = 33) reported mind-wandering during the wakeful rest delay condition, incidentally recalling the past and thinking about the future**.** In the cued retrieval/future imagination delay condition 14 participants predominantly recalled autobiographical memories while the remaining 22 participants predominantly imagined future scenarios. Wordlist retention did not differ significantly between these retrospectively formed ‘memory recall’ and ‘future imagination’ groups (*F*(1,34) = 0.035, *p* = .854, ηρ^2^ = .001).

In the picture search delay condition there were no significant correlations between the level of retention of the associated wordlist and the number of picture details described, neither for the online descriptions given during the 9-minute delay period itself (*R^2^* = .008, *p* = .602), nor for the post-experimental recall of the pictures (*R^2^* = .003, *p* = .754).

In the cued retrieval/future imagination delay condition there were no significant correlations between the level of retention of the associated wordlist and the quantitative internal score for the online descriptions given during the 9-minute delay period itself (*R^2^* = .003, *p* = .732), nor for the post-experimental recall of these cued memories/imaginations (*R^2^* = .002, *p* = .784). This was also true for the quantitative total (internal + external) score for online descriptions during the delay period itself (*R^2^* = .015, *p* = .472), and for post-experimental recall of cued memories/imaginations (*R^2^* = .000, *p* = .960).

However, in the cued retrieval/future imagination condition, there was a significant negative correlation between the level of retention of the associated wordlist and the mean qualitative score of episodic richness of the *online descriptions* given during the 9-minute delay period itself (*R^2^* = .214, *p*<.005). There was no significant correlation between the level of wordlist retention and the mean qualitative score of episodic richness of the *post-experimental recall* of the memories/imaginations that had been cued during the cued retrieval/future imagination delay condition (*R^2^* = .057, *p* = .161). Moreover, there were no other significant correlations between wordlist retention and qualitative Levine sub-scores for descriptions given during the cued retrieval/imagination delay, or during post-experimental recall (all *p*>.223).

The number of details described in the picture search delay condition correlated significantly and positively with the quantitative internal score in the cued retrieval/future imagination delay condition, both during the delay itself (*R^2^* = .324, *p*<.001) and during the post-experimental recall of the pictures/memories/imaginations (*R^2^* = .344, *p*<.001).

Given the counterbalanced nature of the paradigm, the length of the delay interval between the cued retrieval/future imagination condition and delayed word recall/post-experimental recall of previously cued memories/imaginations was not equal across participants (∼27-18 minutes, N = 12; ∼18-9 minutes, N = 12; ∼9-1 minutes, N = 12). However, neither wordlist retention nor post-experimental recall of cued memories/imaginations was affected significantly by these variations in the length of the delay condition – delayed recall test interval. This finding indicates that the above correlations between wordlist retention and descriptions of cued memory/future imagination were not confounded by variations in the length of the interval between the delay condition and delayed recall. The same was true for the picture search delay condition.

#### Comments

Experiment 1 confirms that people retain less newly learned episodic material when the learning period is followed immediately by new external information than when the learning period is followed immediately by wakeful resting [1]–[4], [27]. Importantly, our cued retrieval/future imagination delay condition had an equally detrimental effect on the retention of newly learned episodic material.

The ‘describe aloud’ procedure was chosen as it allowed us to (i) ascertain that participants were performing the cued retrieval/future imagination task, and (ii) quantify their task performance. However, there is the possibility that the verbal description, rather than the cued retrieval/imagination, caused the observed interference effect in the cued retrieval/future imagination delay condition, e.g. due to the need for semantic retrieval and speech production, and/or due to the encoding of articulated, narrated descriptions into episodic memory. The question of whether or not the verbal descriptions produced the interference observed is not only of theoretical interest; it is also of interest to the generalizability of these findings to everyday life, wherein people frequently think about the past and future *in silence*, without the need for rich, verbal descriptions. If the interference observed in the cued retrieval/future imagination delay condition was produced by verbal description, the interference effect should vanish in the absence of a verbal description task. This was the focus of Experiment 2.

## Experiment 2

In Experiment 2 we examined whether the cued memory recall/future imagination task lowers retention of newly learned words, even when no verbal descriptions are required.

### Method

#### Participants

36 new healthy young adults (10 males, 26 females; mean age = 20.2 years, *SD* = 1.99) participated in Experiment 2.

#### Procedure

The procedure was the same as that for Experiment 1 except that participants were asked to perform the cued retrieval/imagination and picture search tasks in silence rather than provide a verbal description.

### Results

#### Immediate word recall

There was no difference between the delay conditions in immediate recall performance (*F*(2,70) = 0.042, *p* = .959, ηρ^2^ = .001), indicating that baseline memory performance was matched across the three delay conditions.

#### Wordlist retention

Mean percentage retention scores ((delayed recall/immediate recall) ×100) are displayed in Figure 2. A main effect of delay condition was observed (*F*(2,70) = 16.967, *p*<.001, ηρ^2^ = .500). Planned *t* tests showed that wordlist retention was significantly higher when learning was followed by wakeful rest than by the picture search (*t*(35) = 2.902, *p*<.05) or cued retrieval/future imagination (*t*(35) = 5.673, *p<*.001) delay conditions. Retention was also significantly higher when word learning was followed by the picture search delay condition than when word learning was followed by the cued retrieval/future imagination delay condition (*t*(35) = −2.235, *p*<.05).

#### Expected recall and rehearsal

Two participants expected the delayed recall and thought about wordlist material during some of the rest delay. However, none of the results changed when removing these participants from the analyses.

#### Delay condition activity and wordlist retention

The majority of participants (N = 34) reported mind-wandering during the wakeful rest delay condition, incidentally recalling the past and thinking about the future**.** In the cued retrieval/imagination delay condition, 17 participants predominantly recalled autobiographical memories while the remaining 19 participants predominantly imagined future scenarios. Wordlist retention did not differ significantly between these retrospectively formed ‘memory recall’ and ‘future imagination’ groups (*F*(1,34) = 0.103, *p* = .750, ηρ^2^ = .003). There were no significant correlations between the level of wordlist retention and the level of post-experimental cued recall of the scenarios, neither in the picture search delay condition nor in the cued retrieval/future imagination delay condition.

### Experiment 1 Versus Experiment 2

#### Wordlist retention

Planned *t* tests showed that retention did not differ significantly between Experiment 1 and 2 in the wakeful resting delay condition (*t*(70) = −0.663, *p* = .510), the picture search delay condition (*t*(70) = −1.555, *p* = .125) or crucially, the cued retrieval/imagination delay condition (*t*(70) = 0.474, *p* = .637). Moreover, there were no significant interactions between Experiment and delay condition when contrasting retention in the wakeful rest delay condition with retention (i) in the picture search delay condition (*F*(1, 70) = .409, *p* = .524, ηρ^2^ = .006) or (ii) in the cued retrieval/future imagination delay condition (*F*(1, 70) = .788, *p* = 0.378, ηρ^2^ = .011).

#### Post-experimental recall of sound-related pictures and cued memories/imaginings

There were no significant differences between Experiment 1 and Experiment 2 in the post-experimental recall of (i) the number of internal details (*t*(70) = 1.310, *p* = .194), (ii) the total number of (internal + external) details (*t*(70) = 1.519, *p* = .133), (iii) the qualitative episodic richness score of previously cued memories/imaginings (*t*(70) = −1.599, *p* = .124), or (iv) sound-related pictures (*t*(70) = 1.749, *p* = .085). This finding suggests that the level of retrieval/imagination and picture viewing during the delay itself was similar in the two experiments, despite the variations in instructions.

#### Comments

Our picture search delay condition, and crucially our cued retrieval/future imagination delay condition interfered with the retention of new episodic memories, irrespective of whether or not people had to verbally describe their memories/imaginations during the delay. This finding rules out a major involvement of verbal description in the interference effect observed in the cued retrieval/future imagination delay condition of Experiment 1, and instead suggests that it was cued retrieval/future imagination per se that interfered with wordlist memory. However, given the presence of sounds in the cued retrieval/future imagination delay, we cannot rule out the possibility that the mere anticipation and/or encoding of these sounds were responsible for the observed interference effect in Experiments 1 and 2. We sought to exclude this possibility via Experiment 3.

## Experiment 3

In Experiment 3 we investigated whether the mere anticipation and/or encoding of our sound stimuli interferes with wordlist retention.

### Method

#### Participants

36 new healthy young adults (19 males, 17 females; mean age* = *20.97 years, *SD* = 2.32) participated in Experiment 3.

#### Procedure

The procedure was the same as that for Experiment 2 except that the cued retrieval/future imagination delay condition was now replaced by a ‘sounds only’ delay condition. In this delay condition we presented the familiar sounds from the cued retrieval/future imagination delay condition of Experiments 1 and 2. However, in contrast to Experiments 1 and 2, participants in Experiment 3 were informed that they should sit and relax and that several sounds would be presented at varied time intervals to minimise the risk of them falling asleep. They were told that they would not be required to perform any task during this period. We also added a question to the post-experimental questionnaire to verify whether the sounds had spontaneously triggered memories/imaginations in any participants.

### Results

#### Immediate word recall

There was no difference between the delay conditions in immediate recall performance (*F*(2,70) = 0.210, *p* = .811, ηρ^2^ = .006), indicating that baseline memory performance was matched across the three delay conditions.

#### Wordlist retention

**Figure 3:**
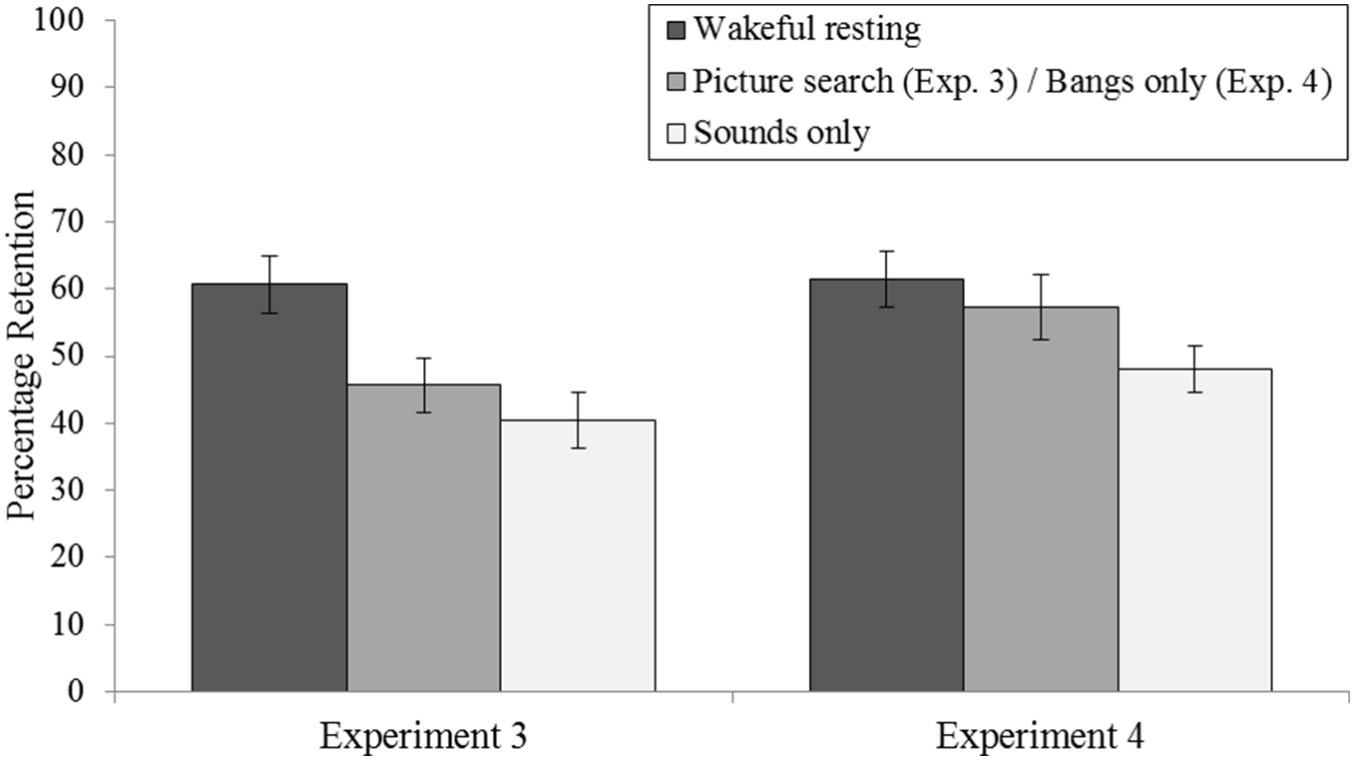
Mean percentage-retention scores as a function of delay condition in Experiment 3 (wakeful resting vs. picture search vs. ‘sounds only’) and in Experiment 4 (wakeful resting vs. ‘bangs only’ vs. ‘sounds only’). All three delay conditions were performed in silence. Participants were not instructed to retrieve memories/imagine future scenarios in any of the delay conditions. Percentage-retention scores were calculated by dividing the number of words recalled correctly in the delayed recall test by the number of words recalled correctly at immediate recall and multiplying by 100. Error bars show the standard error of the mean.

Mean percentage retention scores ((delayed recall/immediate recall) ×100) are displayed in Figure 3. A main effect of delay condition was observed (*F*(2,70)* = *7.156, *p<*.001, ηρ^2^ = .170). Planned *t* tests showed that wordlist retention was significantly higher when learning was followed by wakeful rest than by the picture search (*t*(35) = 4.055, *p*<.01) or ‘sounds only’ (*t*(35) = 2.839, *p*<.001) delay conditions.

#### Expected recall and rehearsal

Two participants expected the delayed recall and thought about wordlist material during some of the rest delay. However, none of the results changed when removing these participants from the analyses.

#### Delay condition activities

The majority of participants (N = 33) reported mind-wandering during the wakeful rest delay condition, incidentally recalling the past and thinking about the future**.** When probed after the experiment, 26 participants reported sound-related memories/future imaginations during the ‘sounds only’ delay condition. The remaining 10 participants reported mind-wandering during the ‘sounds only’ delay condition.

### Experiments 1–3

#### Wordlist retention

Planned *t* tests showed that wordlist retention did not differ significantly between Experiment 2′s cued retrieval/future imagination delay condition and Experiment 3′s ‘sound only’ delay condition (*t*(70) = −.287, *p* = .775). In addition, no significant difference was observed in wordlist retention between the picture search delay condition of Experiments 2 and 3 (*t*(70) = 1.441, *p* = .154), or Experiments 1 and 3 (*t*(70) = −0.047, p = .963).

#### Comments

In contrast to our predictions, the ‘sounds only’ delay condition produced significant levels of interference with episodic memory. At a first glance, this finding suggests that the anticipation/encoding of sounds might have in fact produced the interference observed in the cued retrieval/future imagination delay condition in Experiments 1 and 2, and in the ‘sounds only’ condition in Experiment 3. However, to our surprise, the presented sounds spontaneously triggered memories/imaginations in most participants. This unexpected observation actually fits the finding that external concrete cues, especially sounds, frequently trigger ‘involuntary’ memories [15]. It thus appears likely that this (involuntary) retrieval/future imagination accounted for the interference effect observed in our ‘sounds only’ condition. However, given the presence of sounds *and* (unanticipated) cued retrieval/future imagination in the ‘sounds only’ delay condition, we still cannot rule out the possibility that the mere anticipation and/or encoding of sounds was responsible for the observed memory interference in Experiments 1–3. We re-examined this possibility in Experiment 4.

## Experiment 4

In Experiment 4 we investigated the specific effect of sound anticipation/encoding on episodic memory retention. We did so by utilising meaningless sounds so as to minimise the cueing of involuntary memories/future imaginations that was observed via familiar, concrete sounds in Experiment 3.

### Method

#### Participants

36 new healthy young adults (10 males, 26 females; mean age = 20.4 years, *SD* = 1.78) participated in Experiment 4.

#### Procedure

As in Experiment 3 we included the wakeful resting delay condition and the ‘sounds only’ delay condition. However, we replaced the picture search delay condition with a new ‘bangs only’ delay condition. Here participants were presented with 10 different meaningless “bang” sounds at the same varied time intervals as used across Experiment 1–3. We also added a question to the post-experimental questionnaire to verify whether the “bang” sounds had spontaneously triggered memories/imaginations in any participants.

### Results

#### Immediate word recall

There was no difference between the delay conditions in immediate recall performance (*F*(2,70) = 0.676, *p* = .512, ηρ^2^ = .019) indicating that baseline memory performance was matched across the three delay conditions.

#### Wordlist retention

Mean percentage retention scores ((delayed recall/immediate recall) ×100) are displayed in Figure 3. A main effect of delay condition was observed (*F*(2,70) = 3.317, *p<*.05, ηρ^2^  = .087). Planned *t* tests showed that wordlist retention was significantly higher when learning was followed by wakeful rest than by the ‘sounds only’ delay condition (*t*(35) = 2.545, *p*<.05). There was no significant difference in retention between the wakeful resting delay condition and the ‘bangs only’ delay condition (*t*(35) = .760, *p* = .452). However, retention was higher when learning was followed by the ‘bangs only’ delay than by the ‘sounds only’ delay condition, and this difference was near-significant (*t*(35) = −1.755, *p* = .088).

#### Expected recall and rehearsal

Three participants expected the delayed recall and thought about wordlist material during some of the rest delay. However, none of the results changed when removing these participants from the analyses.

#### Delay condition activities

The majority of participants (N = 33) reported mind-wandering during the wakeful rest delay condition, incidentally recalling the past and thinking about the future. When probed after the experiment, 25 participants reported sound-related retrieval of memories/future imaginations during the ‘sounds only’ delay condition. The remaining 11 participants reported mind-wandering. No participants reported sound-related retrieval of memories/future imaginations during the ‘bangs only’ delay condition.

#### Comments

The results of Experiment 4 suggest that the memory interference observed during cued retrieval/imagination in Experiments 1–3 was not simply the product of sound anticipation/encoding.

### Experiment 1–4

#### Effect of wordlist position and position of the wakeful rest delay condition

Given that retention of the three wordlists was probed within a single test of delayed recall, the delay intervals between learning and delayed recall varied between wordlist 1 (∼27 minutes), wordlist 2 (∼18 minutes) and wordlist 3 (∼9 minutes). We therefore examined whether delay interval length affected retention in our experiments. Across Experiments 1–4 there was no main effect of delay interval length (*F*(2,280) = 1.707, *p* = .182, ηρ^2^ = .012), Experiment (*F*(3,140) = 0.936, *p* = .425, ηρ^2^ = .020), or interaction between delay interval length and Experiment (*F*(6,280) = 1.477, *p* = .186, ηρ^2^  = .031).

In order to ascertain that consolidation, rather than a passive minimal interference effect, was at play in the memory boost via wakeful rest in our paradigm, we examined whether (i) the beneficial effect of wakeful rest was observed independently of the position of the wakeful rest delay condition in the experiment, and (ii) whether there was a temporal gradient of interference [2], [3], [6], [10] across Experiments 1–3. Addressing (i), the degree of benefit from wakeful rest did not differ between the participants who received the wakeful rest delay first, second or third (i.e. no significant interaction between delay condition and position of the wakeful rest delay, *F*(4,210) = 0.399, *p* = .809, ηρ^2^ = .011). In fact, wordlist material learned immediately prior to the wakeful rest delay was retained equally well in participants who received the wakeful rest delay first (68.25%), second (61.10%) or third (66.43%) (i.e. no significant effect of position of the wakeful rest delay, *F*(2,105) = 0.846, *p* = .432, ηρ^2^ = .016). Addressing (ii), wordlist 1 was retained significantly better after the Experiment (after ∼27 minutes) when wordlist 1 learning was followed immediately by the 9-minute wakeful rest delay condition (68.22%), thus delaying the onset of the task-filled delay conditions, than when wordlist 1 learning was followed immediately by a 9-minute task-filled delay (41.83%) (*t*(70) = 4.646 *p*<.001). There was no significant difference in wordlist 1 retention when wakeful resting occurred after a 9-minute (41.83%) or after an 18-minute (48.75%) task-filled delay (*t*(70) = −1.151, *p* = .254). In order to ensure that these effects were not confounded by the position of the wordlist within the experiment, we repeated the same analyses for wordlist 2. The effect of position of wakeful rest was identical: wordlist 2 was retained significantly better after the Experiment (∼18 minutes) when wordlist 2 learning was followed immediately by the 9-minute wakeful rest delay condition (65.66%) than when wordlist 2 learning was followed immediately by a 9-minute task-filled delay (42.25%) (*t*(70) = 4.556, *p*<.001).

## Discussion

Our results demonstrate that, like external information, cued autobiographical memory retrieval and cued imagination of the future interfere with the retention of recently-acquired episodic memories. Across all experiments, participants retained more wordlist material when wordlist learning was followed immediately by wakeful resting than when wordlist learning was followed immediately by retrieval of past memories/imagination of future scenarios, cued by familiar sounds. As with previous research [4] only a few participants in each of our experiments thought about the wordlist material during the rest delay. When these participants were removed from our analyses no differences in results were observed. Thus, it is unlikely that the interference effect in the picture search and cued retrieval/future imagination delays can be accounted for merely by reduced intentional rehearsal/thinking of wordlist material.

It is also unlikely that this interference effect can be accounted for by the mere displacing of recently acquired transient memory traces by subsequent information. This hypothesis of a ‘passive’ effect of wakeful rest posits that the benefit of wakeful rest lasts only until people are exposed to interfering material [2], [10], [28]. The findings of (i) a lasting beneficial effect of wakeful rest after exposure to further material [4] and (ii) a temporal gradient of interference are not in keeping with this interference hypothesis.

This lasting beneficial effect of wakeful rest and the temporal gradient of interference can however be accounted for straightforwardly by memory consolidation. Memory consolidation is defined as the process by which new memories strengthen over time, becoming less susceptible to interference [6], [29]. We thus hypothesise that the interference effect observed in our picture search task and in our cued retrieval/future imagination task was the result of interference with automatic memory consolidation processes [4]. We will return to this hypothesis later in the discussion.

What interfered with consolidation in our cued retrieval/future imagination task? Experiments 1 and 2 showed that the interference could not be accounted for merely by the aloud verbal descriptions provided in Experiment 1: the interference effect in the picture search and cued retrieval/future imagination delay conditions occurred irrespective of whether or not participants had to provide verbal descriptions of the scenarios. In Experiment 2, wordlist retention was in fact higher in the picture search delay condition when undertaken in silence than when accompanied by verbal descriptions (see Figure 2). However, this difference was not statistically significant, and likely the product of some high performing participants in Experiment 2. Indeed, no such increase in retention was observed in the picture search delay condition in Experiment 3, in which picture search was also undertaken in silence (see Figures 2 and 3). More importantly, in the cued retrieval/future imagination delay condition, wordlist retention did not differ between the participants who undertook the task in silence (Experiment 2) and those who were tasked with providing verbal descriptions of their memories/imaginings (Experiment 1). We acknowledge that some participants in Experiment 2 (and in the ‘sounds only’ delay condition of Experiments 3 and 4) may have spontaneously narrated their memories/imaginations via inner speech, and that this inner speech could have produced some specific verbal interference. However, it is unlikely that such inner speech could have been at the heart of the interference observed in the cued retrieval/future imagination delay condition. If mere verbalization of memories/imaginings had contributed substantially to the interference effects observed in the cued retrieval/future imagination delay condition, then one would have expected there to be a negative correlation between (i) the total number of (internal + external) details uttered during the delay itself and (ii) the level of wordlist retention. However, this was not the case (*R^2^* = .015, *p* = .472; Experiment 1). This finding suggests that the interference observed across our Experiments was produced by the cued retrieval/future imagination task itself rather than by potential concurrent verbalisation of what was being retrieved/imagined.

In Experiments 1 and 2 participants were instructed to retrieve autobiographical memories/imagine future scenarios relating to the sounds played. This will have resulted in strategic search and related executive processes [30]. It is unlikely however that such executive processes caused the memory interference observed. In Experiment 3 and 4 the majority of participants reported spontaneous sound-related memories/future imaginings even though they had not been given any instructions to use the sounds as cues (‘sounds only’ condition). Research shows that ‘involuntary’ memories, such as the ones observed in Experiment 3 and 4, require very little executive processing [30]. In fact, external sound cues can even reactivate memories during sleep [31]. Aside from executive processes, voluntary and involuntary memories are said to have a common cognitive basis [30]. The fact that voluntary and involuntary memories/imaginings interfered to the same extent suggests that the interference was produced either by the sounds or by the automatic processing of autobiographical memory traces/imagined scenarios, rather than by executive retrieval mechanisms.

The findings in Experiment 4 suggest strongly that the mere anticipation/encoding of sounds cannot account for the interference observed in our cued retrieval/future imagination condition. When external sound cues were not concrete (‘bangs only’ delay condition), subsequent wordlist retention was equivalent to that seen following wakeful resting. This suggests strongly that some more elaborate cognitive processing must have occurred following sound presentation in order for interference to occur. Indeed, whereas all participants mind wandered in the ‘bangs only’ delay condition (Experiment 4), the majority of participants reported recalling sound-related memories/future imaginations in the ‘sounds only’ delay conditions (Experiment 3 and 4). It could be argued that the observed interference via familiar sound cues was simply associated with covert or overt identification of these sounds, rather than with the processing of memories/imagination of future scenarios. If so, the richness of retrieved memories/future imaginations should not have affected wordlist retention.

However, Experiment 1 revealed that wordlist retention decreased significantly as a function of increasing (qualitative) episodic richness of autobiographical memories/future imaginings. This finding suggests strongly that it was the processing of autobiographical memories/future scenarios that interfered with wordlist retention. Moreover, wordlist retention was not associated with the number of semantic details described, suggesting that interference was particular to the episodic nature of autobiographical retrieval/future imagination.

As indicated in the introduction, the encoding of novel external information is hypothesised to interfere with the automatic offline replay/early (i.e. cellular) consolidation of recently acquired memory traces [4], [10]. Our finding of a temporal gradient of interference bolster this hypothesis and suggest that autobiographical retrieval and future imagining also interfere with early (i.e. cellular) memory consolidation. Like the encoding of external information, the retrieval of autobiographical memories/imagination of future scenarios shares some cognitive and neural networks with consolidation [12], [16], [18], [20], [32]–[34]. This cognitive and neural overlap could be at the heart of the memory consolidation interference observed. However, given that encoding is typically accompanied by retrieval, and retrieval is typically accompanied by encoding, it is difficult to disentangle the contributions of these two episodic memory processes to the interference observed here. There is the possibility that our autobiographical retrieval/future imagination task interfered with wordlist consolidation due to the *reactivation* of memory traces associated with the autobiographical memories/imagined future scenarios. However, it is also possible that our autobiographical retrieval/future imagination task interfered with wordlist consolidation due to the subsequent *encoding* of these autobiographical memories/imagined future scenarios. Indeed, at least some encoding must have taken place in order for participants to remember their memories/imaginings after the experiment. This said there was a stronger association between wordlist retention and (qualitative) episodic richness of memories/imaginations during the delay condition itself than after the experiment. This finding could hint tentatively that the memory interference was produced primarily by the reactivation/reconstruction of past autobiographical memory traces rather than by subsequent encoding of those autobiographical memory traces. However, more extensive work is required to dissociate the specific contributions of encoding and retrieval to consolidation interference.

Irrespective of the specific processes that are responsible for this consolidation interference, the correlational analyses could suggest that memory interference via cued retrieval/future imagination is not associated strongly with the *quantity* of retrieved/imagined information. However, the number of described details might not necessarily reflect the ‘true’ richness of a person’s memory/imagined scenario. Some people might simply provide less detailed descriptions than others, irrespective of the richness of their memories/imagined scenarios [25]. Evidence for this hypothesis comes from the finding of individual differences in the number of details described in the picture search task, in which images were of equal perceptual richness across participants. Moreover, there was a strong correspondence in Experiment 1 between the number of details described in (i) the picture search task, and (ii) the cued retrieval/future imagination task. Therefore, the stronger correlation between wordlist retention and (qualitative) episodic richness than between wordlist retention and number of details described might simply reflect differences in sensitivity to true richness of memory/imagination.

It is of interest that across Experiments 1–4, the majority of participants reported mind wandering during wakeful resting [4], incidentally recalling the past and thinking about the future. It is unclear why participants retained more wordlist material following this retrieval/future imagination during mind wandering than following externally cued retrieval/future imagination. One possibility is that these conditions differed *qualitatively* in that different cognitive processes might have been active during externally and internally cued autobiographical retrieval/future imagining [35], [36]. Alternatively, it is possible that these conditions differed *quantitatively*. Participants might have retrieved more memories/imagined more scenarios when external concrete cues were present than when they were absent, resulting in more interference during the former condition. Indeed, research suggests that involuntary autobiographical memories are more frequently triggered by external cues than by internal cues (thoughts and emotions) [30].

It is also possible that autobiographical memories/imaginings were more complex and episodically-rich when cued externally than internally, thus interfering more with the consolidation of wordlist material. Evidence for this hypothesis comes from the finding that participants whose externally cued autobiographical memories/imaginings were episodically sparse retained more wordlist material than those participants whose externally cued autobiographical memories/imaginings were episodically rich. In the same vein, a participant resting quietly would likely show lowered wordlist retention if internal cues triggered particularly rich autobiographical memories/imaginings immediately after the word learning period. Indeed, this might explain individual differences in the degree of benefit gained from wakeful resting after new learning. Further work should probe in detail what participants thought about during the wakeful rest delay, so as to allow for correlations between the richness of autobiographical thinking and wordlist retention during wakeful rest.

In addition to encoding large amounts of new external information, humans spend a lot of time recollecting their past and thinking about their future, often triggered by external cues. Our study shows that these activities hamper the consolidation of recently acquired episodic memories. Therefore, if one wishes to retain newly acquired information well, it is advantageous to rest for a few minutes after learning, avoiding external information and cues *as well as* complex reminiscing about one’s past and future.
